# Prevalence of hypoxemia among sick children, aged under-five years, seeking healthcare at primary health facilities in Uttar Pradesh, India: an observational-cohort study

**DOI:** 10.1016/j.lansea.2025.100706

**Published:** 2025-12-15

**Authors:** Shally Awasthi, Divas Kumar, Anuj Kumar Pandey, Girdhar Gopal Agarwal, Anmol Jacob, Kovid Sharma, Monika Agarwal, Hélène Langet, Gaurav Kumar, Gillian A. Levine, Silvia Cicconi, Michael Ruffo, Mira Emmanuel-Fabula, Fenella Beynon, Fabian Schär, Kaspar Wyss, Valérie D'Acremont, TIMCI Collaborator Group

**Affiliations:** a Department of Pediatrics, King George's Medical University, Lucknow, India; b Department of Statistics, University of Lucknow, Lucknow, India; c PATH, USA; d Department of Community Medicine, King George's Medical University, Lucknow; e Swiss Centre for International Health, Swiss Tropical and Public Health Institute, Allschwil, Switzerland; f University of Basel, Basel, Switzerland; g Department of Medicine, Swiss Tropical and Public Health Institute, Allschwil, Switzerland; h Global and Environmental Health Sector, Centre for Primary Care and Public Health (Unisanté), University of Lausanne, Lausanne, Switzerland

**Keywords:** Hypoxemia, Pulse oximetry, Prevalence, Sick children, Referral, Rural health facilities, Northern India

## Abstract

**Background:**

Hypoxemia or low blood oxygen saturation (SpO_2_) increases risk of mortality in children aged under-five years. Integrated Management of Neonatal and Childhood Illness guidelines in India recommend referral to higher centre at SpO_2_ <90%. The primary objective was to assess prevalence of hypoxemia among sick under-five, seeking healthcare at rural primary health facilities in Uttar Pradesh, India. Secondary objective assessed completion of referral (defined as reaching higher referral facilities) in hypoxemic children by day-7.

**Methods:**

Secondary data analyses from pulse oximetry (POx) arm of a cluster randomized trial. Hypoxemia, defined as SpO_2_ <94%, was assessed on day-0 and completion of referral through telephonic follow-up on day-7 (+2 days). Severe hypoxemia was defined as SpO_2_ <90%. Registration number: CTRI/2022/03/041325.

**Findings:**

From 20-June-2022 to 21-April-2023, 24,966 children were enrolled, among whom SpO_2_ readings were available for 94·4% (n = 23,560) children. Prevalence of hypoxemia was 1·3% (308/23,560) and severe hypoxemia was 29·2% (90/308). Lost to follow-up among hypoxemic was 9·4% (29/308). Among hypoxemic 27·9% (86/308) and among severe hypoxemic 46·7% (42/90) children were referred. Referral completion among hypoxemic was 20·3% (16/79) and severe hypoxemic 23·8% (10/40). Mortality in hypoxemic children was 3·9% (11/279), vs 0·05% (11/20,292) in non-hypoxemic ones.

**Interpretation:**

Prevalence of hypoxemia is low among sick children presenting to rural health facilities of northern India. Less than one-third of hypoxemic children (and less than half of severely hypoxemic) were referred to higher facility, and only one in five completed referrals. Therefore, identifying and addressing the factors associated with these findings, would strengthen the integration of POx in primary healthcare facilities.

**Funding:**

This work is supported by Unitaid as part of the Tools for Integrated Management of Childhood Illness (TIMCI) project under grant number n°2019-35-TIMCI to PATH.

**Research in context**

**Evidence before this study**

Evidence shows that hypoxemia increases the risk of death in sick children by approximately five times. In resource limited settings detection of hypoxemia is a challenge as there is no single clinical sign that accurately identifies it. Therefore, pulse oximetry (POx) could potentially be useful for the detection of hypoxemia. Studies from low-and-middle-income countries (LMICs) from Africa and Southeast Asia, using POx, have reported a varied range of prevalence of hypoxemia among children presenting at primary healthcare facilities. A meta-analysis, conducted by Rahman et al., reported 31·0% prevalence of hypoxemia across LMICs, among children with WHO-classified pneumonia. A single study from northern India reported 35·9% prevalence of hypoxemia among children hospitalized with community acquired pneumonia. Another, meta-analysis conducted by Graham et al., reported that the pooled prevalence of hypoxemia among hospitalized neonates was 24·5% and 12·1% among children aged 1 month to 17 years from LMICs. There is, however, dearth of data from India about the prevalence of hypoxemia, as POx is not used as a part of routine care at rural primary health facilities, and decisions taken upon detection of hypoxemia by clinicians and caregivers are unknown.

**Added value of this study**

The current study provided data about the prevalence of hypoxemia in children under-five years seeking care at rural primary health facilities due to any illness, in Northern India. The study found low prevalence of hypoxemia using POx, but the mortality was high among hypoxemic children compared to non-hypoxemic children. Unfortunately, less than one-third of hypoxemic children (and less than half of severely hypoxemic ones) were referred to a higher facility by clinicians, and, among them, only one in five could reach it. About half of the deaths among children referred to higher facilities occurred at home. Additionally, Integrated Management of Neonatal and Childhood Illness, in India, recommends urgent referral of only those hypoxemic children who present with cough or difficult breathing. However, evidence from this study shows that even this guideline is not adhered to. Hypoxemia in the sick children is not seasonal; it occurs round the year and in those with any danger sign and fever and not just in those with symptoms of respiratory illness.

**Implications of all the available evidence**

Even if few sick hypoxemic children present at rural health centers in northern India, they are at increased risk of death. Yet not all hypoxemic children are referred to higher facilities by health care providers. Among those referred, the majority of caregivers do not accept referral. Therefore, systems strengthening is needed which should include training on identification of hypoxemic children by health care providers, establishment of effective and timely referral systems, clinical audits and accountability of healthcare providers, and facilitating acceptance of referral by caregivers by incorporating incentives if necessary, and ensuring no out of pocket expenditure. This would involve further system and implementation research followed by dialogues with policymakers and planners. These steps are essential to determine optimal timing for effective introduction of POx at primary healthcare level. Simultaneously, strategies must focus on the improvement of quality of care in the public health system to ensure increased demand. Further, the Integrated Management of Neonatal and Childhood Illness Guidelines, in India, should be revised to include pulse oximetry in cases of danger sign and fever. These will help reducing infant and child mortality rates and achieve the Sustainable Development Goal of good health and well-being.

## Introduction

Hypoxemia, defined as low blood oxygen level, either measured in the blood sample or non-invasively, by an oxygen saturation measurement (SpO_2_) using pulse oximetry (POx), is associated with about fivefold increased risk of death in sick children under-five years.^1^ In addition to respiratory conditions like acute lower respiratory tract infections and asthma, hypoxemia is also common in non-respiratory conditions like meningitis/encephalitis, acute febrile encephalopathy, sepsis and malaria in children and neonatal encephalopathy, prematurity and neonatal sepsis.^2^

Early detection and subsequent oxygen administration, along with specific supportive management, is the key to reducing the risk of hypoxemia related adverse events and mortality. However, In India, Integrated Management of Neonatal and Childhood Illness (IMNCI)^3^ guideline states that SpO2<90% is a sign for urgently referring the patient to a higher center with pre referral treatment, only in those with cough or difficult breathing.

Studies have tried to predict hypoxemia on the basis of clinical signs, but there is no single clinical sign, or even combination of signs, that accurately does so.^4^ Hence, by using clinical signs alone there are chances of either missing cases requiring oxygen administration or potential overtreatment of those not requiring it.^4^

The burden of hypoxemic sick children under five years of age, using POx in primary care settings has been assessed by various studies in low-and-middle-income countries (LMICs) from Africa and Southeast Asia. These studies have reported the prevalence of hypoxemia in primary care settings ranging between 1·3% and 9·3%.^5^, ^6^, ^7^, ^8^, ^9^, ^10^ A meta-analysis, reported that across LMICs, the prevalence of hypoxemia was 31·0%, among children with WHO-classified pneumonia.^11^ Another, meta-analysis of studies from LMICs, reported that the pooled prevalence of hypoxemia among hospitalized neonates was 24·5% and 12·1% among children aged 1 month to 17 years.^12^ Studies from India, reported varied prevalence of hypoxemia among children with acute respiratory infection (ARI). A study reported 0·6% of children with ARI had SpO_2_<90%,^13^ while other reported 11·9%.^14^ Another study from northern India, reported 35·9% children (aged 2–59 months), hospitalized due to community acquired pneumonia, were having hypoxic pneumonia.^15^

However, there is dearth of data from India, on the burden of hypoxemia among sick children, under five years of age, at primary healthcare level. To fill this knowledge gap, we report the findings of a secondary analysis of a larger project ‘The Tools for Integrated Management of Childhood Illness (TIMCI),^16^ in this manuscript. The results of TIMCI study are published elsewhere.^10^ The findings presented here are related to prevalence of hypoxemia and its management at primary health care facilities. The primary objective of this manuscript was to assess the prevalence of hypoxemia among sick children, aged under-five years, seeking healthcare at primary health facilities in rural Uttar Pradesh, India. The secondary objective, was completion of the referral, defined as reaching the higher facility of referral, in hypoxemic sick children on day-7. This study quantifies the burden of hypoxemia, providing the healthcare system with data on the higher number of hypoxemic patients referred from primary care to referral facilities. This information is crucial for prioritizing resource allocation toward capacity building, infrastructural strengthening, timely transportation and ensuring oxygen availability.

## Methods

TIMCI^16^ project was a multi-country pragmatic cluster randomized controlled trial (RCT) in India and Tanzania and quasi-experimental pre-post studies in Kenya and Senegal. In India, TIMCI had two arms. In the control arm, usual care was provided to the sick children at primary healthcare facilities, while in the intervention arm usual care was combined with measurement of SpO_2_ by a POx provided by the project. In this manuscript, we report the findings from the POx intervention arm from India. The results of RCT are published elsewhere.^10^

This study was conducted in Uttar Pradesh (UP), a state in northern India, with a population of 200 million, comprising 16·5% of India's population.^17^ UP has higher mortality rates in neonates, infants and young children as compared rest of the country. On comparing UP vs India for the year 2019–21,^18^ neonatal mortality rate has been reported as 35·7 vs 24·9 per thousand live births,^18^ infant mortality rate of 50·4 vs 35·2 per thousand live births^18^ and under-five mortality rate of 59·8 vs 41·9 per thousand live births.^18^

Primary healthcare in rural UP is delivered through state operated primary health centers (PHC) and community health centers (CHC). PHC^19^ serves a population of approximately 15,000–20,000, offering outpatient services including pediatric care. Typically, a PHC has around six beds. PHC is served by one or two doctors, who are either ‘Bachelor of Medicine and Bachelor of Surgery’ (MBBS) or ‘Ayurveda, Yoga and Naturopathy, Unani, Siddha and Homeopathy’ (AYUSH) and the facility is functional during daytime only. Specialists like pediatricians are not posted at PHCs. CHCs^20^ are larger second level facilities serving a population of 100,000–125,000 persons. They provide outpatient services and host an inpatient ward of 30 beds for hospitalizing sick adults, pregnant women and children. These are typically staffed by five to six doctors, often having specialists like pediatricians, obstetricians and anesthetists.

District Hospital (DH)^21^ is the largest tertiary level facility serving the population of the whole district. DH provides specialized outpatient and inpatient services. The number of beds may range from 100 to 700 depending on the population of the district. DH have specialized wards for hospitalizing sick adults, pregnant women and children. These are typically staffed by 30–35 specialist doctors, like pediatricians, obstetricians, anesthetist and surgeons etc. In the TIMCI study, patients were recruited from PHCs and CHCs and the sick hypoxemic children were referred to the DHs.

The various types of healthcare-providers (HCPs) at these facilities are—doctors, trained nurses and pharmacists. Although patient care is primarily provided by doctors, but sometimes, in their absence, patient care is also provided by trained nurses and pharmacists.

The study was conducted in selected rural PHCs and CHCs of three districts of UP namely Sitapur, Unnao and Deoria. Sitapur (mean elevation above sea level 137 m) and Unnao (mean elevation above sea level 122 m) districts, situated in central UP, were selected for operational feasibility, while Deoria (mean elevation above sea level 76 m) district, situated in eastern UP, was selected based on recommendations from the Department of Medical Health, UP (DMH-UP).

### Selection of facilities and participants

This was a two-step procedure. In the first step, facilities were assessed for eligibility and then were randomized by an independent statistician, to the two study arms. In the next step, sick children under 5 years of age were enrolled. Included in this analysis are those children where SpO2 reading available on the day of enrollment.

#### Facility selection

DMH-UP provided the list of PHCs and CHCs of these three districts. The research team visited the facilities, to assess if they met the study inclusion criteria which were: (a) providing curative primary care to children under-five years, (b) availability of electricity and (c) access to medical oxygen, either at same facility or at referral facility, for the management of hypoxemia. Exclusion criteria were (a) less than 20 consultations per month of sick children, (b) pre-existing use of POx during consultation and (c) other research intervention planned during study period. Of 255 facilities assessed, 177 were found eligible from which 40 were randomly selected per arm, as per the requirement of TIMCI.^16^ However, three months later, due to lower than anticipated recruitment rate, the number was increased to 53 (33 PHCs, 20 CHCs) in intervention arm and to 52 (31 PHCs, 21 CHCs) in control arm, using the random selection method as employed earlier.

#### Participant selection

Sick children registering at the selected facilities were screened for eligibility. The inclusion criteria were (a) age under-five years, (b) attending facility for any illness (reported ill by the caregiver) and (c) written voluntary informed consent provided by parents for participation in the study. Exclusion criteria were (a) children in first day of life, (b) already admitted into the facility, (c) attending for trauma or immunization only and (d) previously enrolled into the study within the last 28 days.

Pediatric and neonate appropriate handheld “Acare AH-MX” pulse oximeter, provided by the TIMCI project was used to measure SpO_2_ in all sick children under 5 years. It was selected because it was reported to be reliable, portable, affordable and appropriate for neonates and children.^16^^,^^22^ Three different probes–universal probe, pediatric probe and neonatal wrap, were supplied.

A cascade training approach was used. The central research team in India was trained by the experts from PATH, on use of POx. The central research team then conducted one to one in person training of all the doctors at selected facilities, who provided care to sick children under-five years. Training of the doctors was conducted at their respective health facility. Doctors were trained on the selection of appropriate probe according to the age and size of the participant. The recommendation was to use the probe which ensured adequate fit either to index finger or foot, so that a good waveform was obtained. Doctors were also advised to obtain the reading within 5 min as most readings are obtainable within this time.

In addition, online training of IMNCI^3^ was provided to all the medical doctors by the faculty members of the department of Pediatrics, King George's Medical University, Lucknow, India (KGMU).

Central research team in India, consisted of investigators from KGMU (SA, DK, MA, GGA), investigator from Swiss TPH (GK) and PATH (KS), who were medical doctors with more than ten years of experience in community-based health research. Central coordination was assured by Swiss TPH who did site visits and weekly progress monitoring calls. The site PI and PATH regularly updated the DMH-UP about the progress of the project and its findings.

The field team for the study consisted of Research Assistants (RA), one per selected health care facility for data collection, Field supervisor (FS), one per 4–5 RA for supportive supervision and quality assurance and one District Supervisor (DS) per district, for supportive supervision, quality assurance and a link between the central coordinating team and field team in their respective district.

DS were postgraduates in public health with at least five years of experience in managing community health research projects. FS were postgraduates in science with at least three years of experience in community health research project management and RAs were graduates in science with at least one year of experience in community health research projects.

Members of the field team received four-day in-house training at the coordinating center KGMU, by study investigators on the study protocol,^16^ ICH-GCP guidelines, standard operating procedures, data collection instruments and troubleshooting with the pulse oximeter. After the in-house training, teams were also given practical, hands-on training at the PHCs and CHCs for two days to understand the flow of patient and the processes at the facilities.

**Fig. 1:**
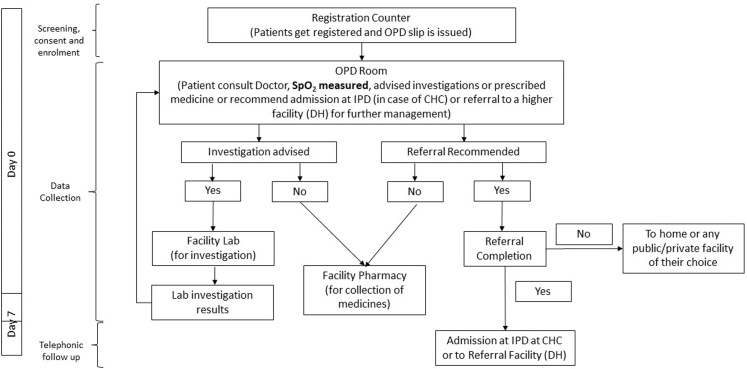
**Flow and scope of TIMCI project: usual path of patient’s journey at rural public health facilities**. OPD: outpatient department, IPD: inpatient department, CHC: community health centre, DH: district hospital.

One RA was placed at each facility for recruitment of study participants. The flow of TIMCI project is given in Fig. 1.

Since the facility registry records limited routine data, a ‘TIMCI consultation card’ was developed by the study and used (Supplementary Figure S1). This recorded demographic, anthropometric variables, vital signs, SpO_2_, diagnosis made by the physician, investigations advised, treatment and management, including referral to a higher center.

After the OPD consultation, the RA recorded socio-demographic details and presenting symptoms by interviewing the caregivers. Other key characteristics required for the study were captured from TIMCI consultation card, which was filled by the consulting doctor. This card also acted as a source document during quality assurance visits.

At day 0 (day of enrollment), the RA met the caregivers of sick under-five children at the registration desk of the facility and assessed for inclusion/exclusion criteria of the index child. If found eligible and consent was obtained from the caregiver, the participant was enrolled into the study. The RA then accompanied the enrolled children throughout their journey in the facility, one sick child at a time.

Once the RA completed the data collection of the first participant then he moved back to the registration desk for the screening and enrollment of next participant. Therefore, RAs could miss some potential participants if accompanying a sick child. Information on the number of missed potential participants was collected. Severely ill children who were taken for emergency care either by the caregivers or hospital staff were not screened. There was, however, no formal system of triage at the facilities.

In TIMCI project, hypoxemia was categorized as severe (SpO_2_ <90%), moderate (SpO_2_ 90–91%), mild (SpO_2_ 92–93%) while no hypoxemia corresponded to a SpO2 ≥94%.^10^^,^^16^ Based on the decision of the doctor in charge, those requiring management at higher level were either admitted at the inpatient ward of CHC or referred to the DH.

A telephonic follow-up of the enrolled sick children was done by contacting the caregivers by the RA at day 7 (+2 days) after enrollment. Information on completion of referral was collected along with the health status of the child. Whenever, the consulting doctor advised the caregivers to get the child admitted at IPD of CHC or visit the DH for further management, the child was classified as having a recommendation for referral. This recommendation was noted in the TIMCI consultation card on Day 0. As a result of the recommendation for referral, those who reached the CHC or DH between day 0 and day 7, were categorized as having completed the referral. Whenever a child got admitted either at CHC or DH, as a result of the recommendation of referral, the event was recorded as “hospitalization”.

Health status on follow-up was recorded as (a) completely cured when the episode of disease for which the consultation had taken place at day 0 had completely resolved; (b) recovering (at home) when the episode of disease for which consultation had taken place at day 0 had not completely resolved but the caregivers reported improvement in child's condition and the child remained at home; (c) hospitalized when the episode of disease for which consultation had taken place at day 0 had not completely resolved and the child was still in the hospital; (d) Death when the child had expired. Any care seeking from private/public health facility between 0 and 7 days after the day 0 consultation, was also recorded.

Standard operating procedures (SOPs) were developed for training, recruitment and follow-up, data capture and data sharing of de-identified data with the Swiss TPH for centralized data cleaning and analysis. Robust monitoring mechanisms were put in place to assure the SOPs were followed. For this, FS visited every facility at least twice a week to observe compliance with SOPs for the data collection and source data verification. Similarly, DS also visited every facility once a month to oversee the functioning of FS and RA. On identification of any deviation, re-training was given to the concerned RA. At the coordinating unit level, data was reviewed weekly for incompleteness, inconsistency, improper format, and duplicity and missing values. In case of any discrepancy, data clarification forms were issued to FS, who then rectified it referring to source data.

The data was collected by the RA using electronic customized case record forms (eCRFs), developed in ODK software. The eCRF was installed in ODK Collect APP on a Samsung Galaxy A tablet which had internet connectivity. The data was synched to a secured, centralized study specific server, located in India. The data was stored in encrypted form and weekly backups were maintained. Data from this server was accessible to the data management team in India.

### Statistical analysis

For the analysis, only those children with SpO_2_ reading available were included. Descriptive statistics for socio-demographic and clinical characteristics of children and type of HCPs consulted, were calculated by facility type. Number and proportions are reported for categorical variables and mean and standard deviations and median and interquartile range (IQR) for continuous variables.

The point prevalence with 95% confidence interval (CI) of hypoxemia was calculated for all included sick children and also by stratum such as socio-demographic and clinical characteristics and type of HCPs consulted.

Descriptive statistics were used to summarize the findings. Adherence to IMNCI guidelines was evaluated based on the proportion of sick children with cough or difficult breathing with SpO2 <90% referred to higher facility for management.

For the analysis of the outcome of referral at day 7, only those with successful day 7 follow-up were included. Health status of the child was also recoded. Health status at day 7 were compared by hypoxemia status at the enrollment, among those who completed or did not complete referral, using the chi-square test. A two-tailed distribution was used, and a p-value of <0·05 was considered statistically significant.

Associations between hypoxemia and clinical characteristics of the children were assessed using multivariable backward stepwise logistic regression analysis. Independent variables with a univariable association with hypoxemia at a two-tailed p-value ≤0·1 were included in the regression model to control for potential confounders. The final model was used to estimate adjusted odds ratio (aORs), adjusted for sociodemographic variables (age, gender), clinical characteristics (duration of illness, presenting symptoms, and danger signs) and health care provider type. Both unadjusted odds ratios (ORs) from the univariable and adjusted odds ratios (aORs) from the multivariable model, along with their 95% confidence intervals, were reported. The risk ratio (RR) with 95% CI was calculated to evaluate the association between hypoxemia and risk of death by day 7.

We also assessed the distribution of hypoxemia by month to observe variations across months of the year. The chi-square test for trend was used to assess association across months. Data were analyzed using SPSS version 24.^23^

### Ethics approval

This study was part of a larger project ‘TIMCI’,^16^ conducted after obtaining ethical approval from WHO Ethics Review Committee (approval number: ERC.0003405, v2·4; dated 02 February 2023) and the Ethics Committee, King George's Medical University (KGMU) (approval number: 1272/ethics/2020; dated 07 December 2020). Written informed consent was obtained from parents of participating children.

### Role of funding source

The funding agency has no role in planning, data collection, analysis and publication of this research.

## Results

**Fig. 2:**
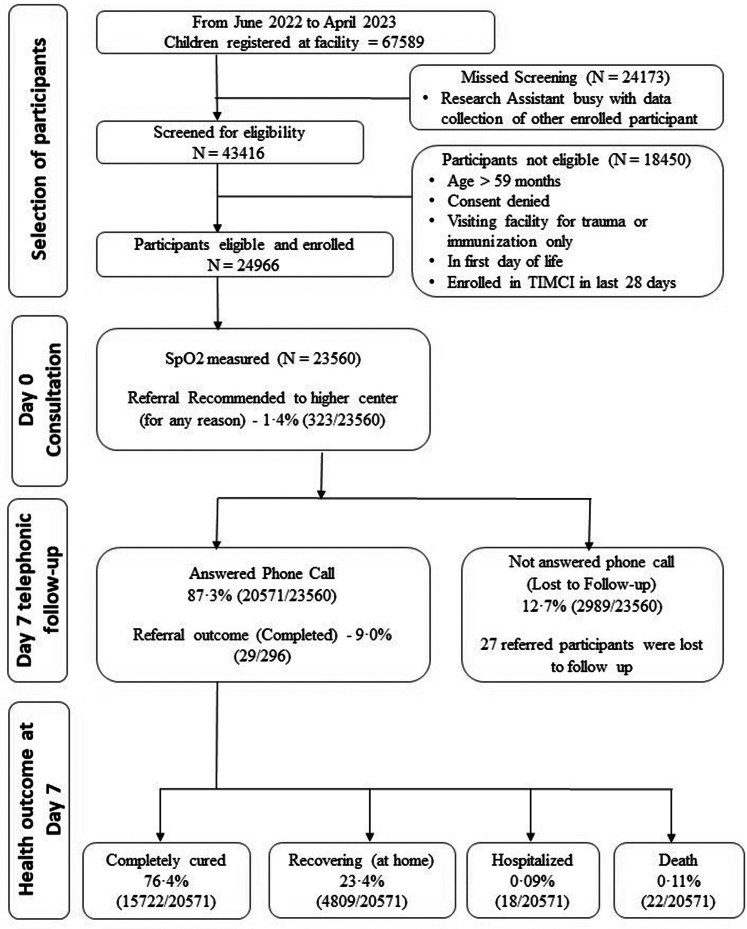
**Flow chart of the participant selection, hypoxemia status and referral recommendation at day of enrolment, follow-up and health outcome at day 7**.

Data was collected from 20 June 2022 to 21 April 2023 from 53 facilities. A total of 24,966 participants were enrolled, 33·5% from PHC and 66·5% from CHCs. Fig. 2 shows the flow chart of participant enrollment, day 0 assessment and follow-up at day 7.

SpO_2_ reading value was available for 94·4% (n = 23,560/24,966) children, of which 93·7% (n = 22,069) were aged 2–59 months and 45·0% (n = 10,592) were females. The median age of sick children was 23·0 months (IQR: 9·0–38·0). Table 1 shows the socio-demographic and clinical characteristics of patients and type of HCPs consulted by them on day 0, distributed by facility type. More than half the children visited the health facility within the first three days after the onset of illness. The most common presenting symptoms were fever (66·8%) followed by cough (44·2%) and runny nose (24·3%). Any danger sign was reported in 3·5% and difficult breathing in 0·7% of the patients. Most of them consulted MBBS doctors (56·2%), followed by AYUSH (20·4%) and Pediatrician (11·0%). The remaining patients were consulted by pharmacists and nurses. There was no difference in the distribution of presenting symptoms of participants, on the basis of type of healthcare provider consulted (Supplemental Table S1).

**Table 1:** Distribution of socio-demographic and clinical characteristics of patients and type of healthcare provider consulted by facility type.

Characteristics of variable	Overall (N = 23,560)	PHC (N = 7992)	CHC (N = 15,568)
	n (%)		
*Age category*			
Infants (under 2 months)	1491 (6·3)	322 (4·0)	1169 (7·5)
Children (2–59 months)	22,069 (93·7)	7670 (96·0)	14,399 (92·5)
*Gender*			
Male	12,968 (55·0)	4376 (54·8)	8592 (55·2)
Female	10,592 (45·0)	3616 (45·2)	6976 (44·8)
*Duration of illness*			
0–3 days	13,253 (56·3)	5220 (65·3)	8033 (51·6)
4–7 days	6703 (28·5)	1811 (22·7)	4892 (31·4)
8–14 days	3604 (15·3)	961 (12·0)	2643 (17·0)
*Presenting* symptoms (multiple choice)			
Any danger sign	822 (3·5)	323 (4·0)	499 (3·2)
Difficult breathing	176 (0·7)	74 (0·9)	102 (0·7)
Fever	15,730 (66·8)	5183 (64·9)	10,547 (67·7)
Cough	10,412 (44·2)	3467 (43·4)	6945 (44·6)
Runny nose/cold	5548 (24·3)	1811 (23·5)	3737 (24·7)
Diarrhea	1222 (5·2)	396 (5·0)	826 (5·3)
Vomiting	1186 (5·0)	402 (5·0)	784 (5·0)
Skin problem	2211 (9·7)	835 (10·8)	1376 (9·1)
Other	2586 (11·0)	576 (7·2)	2010 (12·9)
Healthcare provider type			
Pediatrician	2596 (11·0)	72 (0·9)	2524 (16·2)
MBBS	13,229 (56·2)	3122 (39·1)	10,107 (64·9)
AYUSH	4809 (20·4)	2805 (35·1)	2004 (12·9)
Pharmacist	1841 (7·8)	1578 (19·7)	263 (1·7)
Nurse	1085 (4·6)	415 (5·2)	670 (4·3)

Abbreviations: CHC: Community Health Centre; PHC: Primary Health Centre; MBBS: Bachelor of Medicine and Bachelor of Surgery; AYUSH: Ayurveda, Yoga and Naturopathy, Unani, Siddha and Homeopathy.

Among those without SpO_2_ readings (n = 1406), 93·5% (n = 1315) were aged 2–59 months, 43·3% (n = 609) were female, and 3·7% (n = 52) had any danger sign.

### Prevalence of hypoxemia

The median (IQR) of POx readings was 98% (IQR: 97–98). SpO_2_ cutoff of 94% (corresponding to cutoff of mild hypoxemia) was at 2·5 percentile, SpO_2_ of 92% (corresponding to cutoff for moderate hypoxemia) was at 0·9 percentile and SpO_2_ of 90% (corresponding to cutoff of severe hypoxemia) was at 0·5 percentile.

The prevalence of hypoxemia and its severity level, as well as non-hypoxemia across age group, gender, duration of illness, presenting symptoms and HCP type consulted, along with univariable and multivariable association between hypoxemic and non-hypoxemic children is given in Table 2. The prevalence of hypoxemia was 1·3% (308/23,560) (95% CI 1·2–1·5). In unadjusted analysis, children under 2 months, those with illness duration of more than 3 days, presence of any danger sign, fever, cough, runny nose, diarrhea, skin problem, and type of healthcare provider consulted were significantly associated with hypoxemia. In multivariable two-stepwise model, the odds of hypoxemia were significantly higher in children below 2 months as compared to older ones (aOR 3·64, 95% CI 2·58–5·14). There was no difference in prevalence of hypoxemia by gender. The largest proportion of participants with hypoxemia had illness duration from 4 to 14 days at the time of presentation and the adjusted odds of hypoxemia with duration of illness for 4–14 days was 1·36 times that among those with illness of shorter duration (≤3 days) (95% CI 1·07–1·74). The prevalence of hypoxemia was highest among those presenting with difficult breathing and any danger sign, following by cough, fever, and runny nose and was significantly associated with increased odds of hypoxemia. The proportion of children with hypoxemia detected was highest among those consulted by pharmacists [3·3%, 95% CI 2·5–4·2%), aORs 3·91, 95% CI 2·84–5·37].

**Table 2:** Point prevalence of hypoxemia and its severity level as well as non-hypoxemia along with univariable and multivariable association between hypoxemic and non-hypoxemic enrolled patients seeking care at public health facilities.

Characteristics of variables Row percentage	Hypoxemic status (N = 23,560)		Unadjusted OR (95% CI), p-value^a^	Adjusted OR (95% CI), p-value^a^	Severity of hypoxemia (N = 308)		
	No hypoxemia (N = 23,252)	Hypoxemia (N = 308)			Severe (N = 90)	Moderate (N = 72)	Mild (N = 146)
	n, prevalence (95% CI)				n, prevalence (95% CI)		
*Age category*							
Children (2–59 months) (N = 22,069)	21,811, 98·8% (98·7–99·0)	258, 1·2% (1·0–1·3)	Reference	Reference	79, 0·4% (0·3–0·5)	56, 0·3% (0·2–0·33)	123, 0·6% (0·5–0·7)
Infants (under 2 months) (N = 1491)	1441, 96·6% (95·6–97·5)	50, 3·4% (2·4–4·3)	2·93 (2·16–3·99), **p < 0·001**	3·64 (2·58–5·14), **p < 0·001**	11, 0·7% (0·3–1·2)	16, 1·1% (0·5–1·6)	23, 1·5% (0·9–2·2)
*Gender*							
Male (N = 12,968)	12,788, 98·6% (98·4–98·8)	180, 1·4% (1·2–1·6)	Reference	–	53, 0·4% (0·3–0·5)	40, 0·3% (0·2–0·4)	87, 0·7% (0·5–0·8)
Female (N = 10,592)	10,464, 98·8% (98·6–99·0)	128, 1·2% (1·0–1·4)	0·87 (0·69–1·09), p = 0·23	–	37, 0·4% (0·2–0·5)	32, 0·3% (0·2–0·4)	59, 0·6% (0·4–0·7)
*Duration of illness (in days)*							
0–3 (N = 13,253)	13,098, 98·8% (98·6–99·0)	155, 1·2% (0·9–1·4)	Reference	Reference	45, 0·3% (0·2–0·4)	31, 0·2% (0·15–0·3)	79, 0·6% (0·5–0·7)
4–14 (N = 10,307)	10,154, 98·5% (98·3–98·7)	153, 1·5% (1·3–1·7)	1·27 (1·02–1·59), **p = 0·04**	1·36 (1·07–1·74), **p = 0·01**	45, 0·4% (0·3–0·6)	41, 0·4% (0·3–0·5)	67, 0·7% (0·5–0·8)
*Presenting symptoms*							
Any danger sign (N = 730)	683, 93·6% (91·5–95·2)	47, 6·4% (4·7–8·2)	5·95 (4·32–8·19), **p < 0·001**	4·45 (3·03–6·55), **p < 0·001**	10, 1·4% (0·6–2·5)	19, 2·6% (1·6–4·0)	18, 2·5% (1·5–3·9)
Difficult breathing (N = 176)	136, 77·3% (70·4–83·2)	40, 22·7% (16·5–28·9)	25·37 (17·48–36·82), **p < 0·001**	12·23 (7·58–19·71), **p < 0·001**	8, 4·6% (1·5–7·6)	13, 7·4% (3·5–11·3)	19, 10·8% (6·2–15·4)
Fever (N = 15,730)	15,504, 98·6% (98·4–98·7)	226, 1·4% (1·3–1·6)	1·38 (1·07–1·78), **p < 0·01**	–	65, 0·4% (0·3–0·5)	54, 0·34% (0·25–0·4)	107, 0·7% (0·6–0·8)
Cough (N = 10,412)	10,212, 98·1% (97·8–98·3)	200, 1·9% (1·7–2·2)	2·37 (1·87–2·99), **p < 0·001**	1·99 (1·52–2·59), **p < 0·001**	52, 0·5% (0·4–0·6)	47, 0·5% (0·3–0·6)	101, 1·0% (0·8–1·2)
Runny nose/cold (N = 5548)	5451, 98·3% (97·9–98·6)	97, 1·8% (1·4–2·1)	1·73 (1·35–2·22), **p < 0·001**	1·46 (1·11–1·92), **p = 0·007**	17, 0·3% (0·2–0·5)	21, 0·4% (0·2–0·5)	59, 1·1% (0·8–1·3)
Diarrhea (N = 1222)	1214, 99·3% (98·7–99·7)	8, 0·7% (0·2–1·1)	0·48 (0·24–0·98), **p = 0·04**	–	1, 0·1% (0–0·2)	2, 0·2% (0–0·4)	5, 0·4% (0·1–0·8)
Vomiting (N = 1186)	1174, 99·0% (98·2–99·5)	12, 1·0% (0·4–1·6)	0·76 (0·43–1·36), p = 0·36	–	3, 0·3% (0–0·5)	2, 0·2% (0–0·4)	7, 0·6% (0·2–1·0)
Skin problem (N = 2211)	2197, 99·4% (98·9–99·7)	14, 0·6% (0·3–0·9)	0·50 (0·29–0·86), **p = 0·01**	–	4, 0·2% (0–0·4)	5, 0·2% (0·03–0·4)	5, 0·2% (0·03–0·4)
Healthcare provider type consulted							
MBBS (N = 13,229)	13,079, 98·9% (98·7–99·0)	150, 1·1% (1·0–1·3)	Reference	Reference	20, 0·15% (0·09–0·23)	36, 0·3% (0·2–0·4)	94, 0·7% (0·6–0·9)
Pediatrician (N = 2596)	2574, 99·2% (98·7–99·5)	22, 0·9% (0·5–1·3)	0·75 (0·48–1·17), p = 0·20	0·68 (0·42–1·06), p = 0·09	4, 0·2% (0·04–0·4)	7, 0·3% (0·1–0·6)	11, 0·4% (0·2–0·8)
AYUSH (N = 4809)	4739, 98·5% (98·2–98·9)	70, 1·5% (1·1–1·8)	1·29 (0·97–1·71), p = 0·08	0·86 (0·61–1·22), p = 0·39	36, 0·7% (0·5–1·0)	13, 0·3% (0·1–0·5)	21, 0·4% (0·3–0·7)
Pharmacist (N = 1841)	1780, 96·7% (95·8–97·5)	61, 3·3% (2·5–4·2)	2·99 (2·21–4·04), **p < 0·001**	3·91 (2·84–5·37), **p < 0·001**	30, 1·6% (1·1–2·3)	15, 0·8% (0·5–1·3)	16, 0·9% (0·5–1·4)
Nurse (N = 1085)	1080, 99·5% (98·9–99·9)	5, 0·5% (0·02–0·1)	0·40 (0·17–0·99), **p = 0·047**	0·43 (0·16–1·16), p = 0·09	0, 0·0% (0·0–0·0)	1, 0·1% (0·0–0·5)	4, 0·4% (0·1–0·9)

MBBS: Bachelor of Medicine and Bachelor of Surgery; AYUSH: Ayurveda, Yoga and Naturopathy, Unani, Siddha and Homeopathy.

Bolded p values indicate statistical significance (p < 0·05).

a Unadjusted and Adjusted Odds Ratio was calculated for the variables associated with hypoxemic status.

**Fig. 3:**
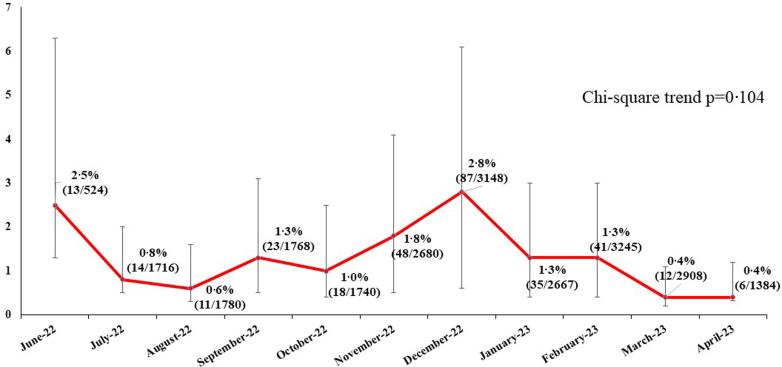
**Month-wise prevalence of hypoxemia**.

District Unnao had the highest proportion of hypoxemic children [1·7% (132/7872)] followed by Sitapur [1·5% (127/8253)] and Deoria [0·7% (49/7435)] (p < 0·001). Fig. 3 shows the month wise variation of prevalence of hypoxemia across all study sites. While the proportion of hypoxemic children presenting to the health facilities was highest during the winter months (November–January), there was no association across the months (p_trend_ = 0·104).

### Referral recommended by health workers and completed referrals

At day 0, HCPs recommended referral for 1·4% (323/23,560) of children, of these 91·6% (296/323) were followed up at day 7. The referral recommendation could be completed only for 9·0% (29/296) children. Among the hypoxemic children referred [27·9% (86/308)], 91·9% (79/86) were followed up at day 7. Of these, 20·3% (16/79) completed the referral. Among children with severe hypoxemia, 46·7% (42/90) were proposed referral by HCPs, and 23·8% (10/40) completed the referral.

### Outcome at day 7

On day 7, 87·3% (20,571/23,560) children were telephonically followed up. Of these, 0·11% (22/20,571) had died. Among the successful follow up of children whose referral was recommended at day 0, 4·4% (13/296) deaths were reported: 6 occurred at home, 6 in the hospital, and 1 during the transit. The prevalence of death in hypoxemic children was 3·94% (11/279), and in non-hypoxemic was 0·054% (11/20,292). The risk ratio of death in hypoxemic children was 72·7 (95% CI 31·8–166·4) as compared to non-hypoxemic.

Among cases with severe hypoxemia (n = 90), 55 children presented with cough and/or difficult breathing. Of these, 54·5% (30/55) were referred on day 0 and of this 28 could be followed up on day 7. Only 30·0% (9/30) had effectively consulted at a referral facility. Among the 9 children who completed the referral, one death was reported.

On day 7, 90·6% (279/308) hypoxemic were followed up. Of these, deaths were reported in 31·3% (5/16) of those who completed referral, 7·9% (5/63) who didn't complete referral and 0·5% (1/200) among those who were not referred, and the difference was statistically significant (p < 0·001).

## Discussion

We found that overall prevalence of hypoxemia (SpO_2_ <94%) was lower than 2% among these children. The prevalence of hypoxemia was 3 times higher in younger (<2 months) compared to older children (between 2 and 59 months). A larger proportion of hypoxemic children sought consultation for symptoms such as any danger sign and difficult breathing compared to non-hypoxemic children. The risk of death were 73 times higher in hypoxemic than non-hypoxemic children.

This study was conducted at rural public health facilities in three districts of UP. These facilities are the first point of care for the rural population. A pediatric appropriate hand-held battery-operated POx (Acare AH-MX) was provided to doctors at these facilities through the project as it provides reliable data in the community settings in previous studies.^22^ Doctors were adequately trained about the usage and maintenance of these devices.

The prevalence of hypoxemia found in present study is lower than the findings of a study from neighboring country Bangladesh which reported 10·6% (SpO_2_ <94%) prevalence of hypoxemia, with 2·7% having SpO_2_ <90%, among children aged 3–35 months visiting the outpatient clinic of Upazila health complex.^8^ Studies from other LMICs like India, have reported varied prevalence of hypoxemia. A meta-analysis, from LMICs, reported that the prevalence of hypoxemia was 31·0%, among children with WHO-classified pneumonia.^11^ Another, reported 24·5% pooled prevalence of hypoxemia among hospitalized neonates and 12·1% among children aged 1 month to 17 years.^12^ A study from Papua New Guinea, reported 3·8% prevalence of hypoxemia (SpO_2_ <94%) with 1·4% having SpO_2_ ≤90%, among children aged 3–27 months visiting rural outpatient clinics.^6^ A recent study from Kenya and Senegal, also reported a higher prevalence of hypoxemia (SpO_2_ <94%) of 5·4–5·8% among children aged up to 59 months, visiting primary care facilities, with 0·7–1·2% participants having SpO_2_ <90%.^9^ The variations can be explained by either variation in the age distribution, the clinical presentation, especially severity or duration of illness as all of these have been found to be associated with increased odds of hypoxemia in the current study.

In India, there is a private as well as public sector providing health care. The current study was restricted to the public sector of UP. Studies in the similar settings from India have reported community's preference for private HCPs and hospitals over public healthcare services.^24^ Hence, fewer severely sick infants and children may have consulted the public primary care health facilities, and this could be another possible reason for low prevalence of hypoxemia found by us. Our findings of low prevalence of hypoxemic children in rural health facilities could be valid and reliable as decrease in prevalence of ARI has been reported by National Family Health Survey (NFHS)-5 (2019–21), compared to NFHS-4 (2015–16), in UP (from 4·7% to 3·5%).^18^ Similarly, there is decrease in percentage of children having fever or symptoms of ARI (from 71·3 to 63·0%) in UP.^18^ This could be the result of the introduction of pneumococcal conjugate vaccine (PCV)-13 in the universal immunization program. In the first phase, PCV-13 was introduced in 19 districts of UP in 2017, which later covered all the 75 districts of UP.

We observed district wise variation in hypoxemia, with lowest prevalence in Deoria and highest in Unnao. Reasons for this may include the care seeking patterns, distance of facility from the homes or severity of sickness in the child. Moreover, due to high incidence of Japanese Encephalitis, Deoria and adjacent districts were under focus of the state government since last eight years, so perhaps there was better care seeking as a result of health facility strengthening. It is pertinent to note that Deoria had the least elevation above sea level among the study districts. However further research is needed to assess district wise incidence of hypoxemia and monitor it longitudinally.

Several studies^25^ have found higher prevalence of hypoxemia during winter season, in line with the finding of current study. We could not find any study from India which had assessed the contributing factors for seasonal variation, but the probable reasons to this include an increase in respiratory tract infections, colder temperatures, and more time spent indoors.^25^ This suggests that the healthcare systems should strengthen the screening through POx and their capacity to either administer oxygen or refer the child with oxygen during the transit. Further studies are required from India to explore the contributing factors and trends of seasonal variation of hypoxemia.

Interestingly, the proportion of hypoxemia reported in sick children varied by the type of HCPs. Among the providers who were trained on POx (Pediatricians, MBBS and AYUSH), there was little variation, however among those who were not trained (pharmacist and nurses) there was a large variation in the detection rate of hypoxemia. This could be a true finding or due to improper use of POx by the untrained HCPs. Further research is needed in this area.

It is concerning to note that only less than one-third of hypoxemic children were referred to the DH by the consulting HCP. This could possibly be due to the IMNCI recommendation of referring only those presenting with cough or difficult breathing and having SpO_2_<90%.^3^ In current study we observed that the odds of hypoxemia among children presenting with danger signs are also high along with those presenting with respiratory symptoms. Children presenting with danger signs and respiratory symptoms should be prioritized for pulse oximetry.

If referral is based on the SpO_2_ reading alone, cutoffs of <94% and <90% would translate into 2·5% and 0·5%, respectively, of sick children being referred to higher centers. This could raise concerns about the capacity of DH to handle additional patient burden with the current infrastructure. Alternatively, HCPs are perhaps trusting clinical judgement over POx reading for referral. Their decision could also be influenced by the preference of the caregivers. Further research is needed to identify reasons for low referral so that they can be addressed. We observed poor adherence to the IMNCI guidelines as only half of severely hypoxemic children with cough or difficult breathing were referred to a higher center for further management. A policy of incorporation of clinical decision support algorithms for management of sick children, in resource limited settings, such as this, could enhance adherence to guidelines and subsequently improved health outcomes.^26^

We found poor completion of referral advice. Only one-fifth of the hypoxemic participants reached the referral facility. Other studies from northern India, having settings similar to that of current study, have reported similar findings. A study from Haryana^27^ and another from Delhi, India^28^ reported poor compliance to referral advice. This may be due to affordability, accessibility and acceptability issues.^28^ Other reasons for non-adherence to referral advice include, illiteracy, absence of male decision-maker, loss of earnings, responsibility of other children and family members, more trust on local doctors and community influence.^27^^,^^28^ Future research is needed in this area.

A telephonic follow-up was done at day 7 to know the clinical outcome of the child. The majority of participants had recovered completely and 0·11% were reported died.

Building on the findings of current study and existing literature^29^ that highlights the need for system strengthening, dialogues with policymakers and planners are essential to determine the optimal timing for introduction of POx at primary healthcare level. Simultaneously, policy must focus on the improvement of quality of care, so that there is an increase in trust of caregivers on the primary healthcare system. Strengthening the referral pathways and incentivizing the families to accept referral is also recommended.

The strength of this study lies in the use of pediatric appropriate POx and data collection round the year to identify seasonal variation, if any. Stringent quality control procedures were in place to ensure validity of the results. In current study, the overall loss to follow-up was 12·7%, and among hypoxemic children it was 9·4%, suggesting that missing data unlikely to affect the representativeness of the sample or the generalizability of the results. The study was done in the public health facilities as the results would feed into policy and facilitate implementation of POx. By this approach, however, we missed assessing the prevalence of hypoxemia in the private facilities, which in any case are very diverse and spread out. One limitation was that one RA was allocated per facility, so there could be a chance of having missed some children, who presented to the facility when the RA was already enrolling or following another patient. The sickest patients were taken for immediate care and were not enrolled in this study, for ethical reasons, which might have led to a lower prevalence than the true prevalence in primary care settings. Since POx was not a part of usual practice, it could have been another reason for low prevalence of hypoxemia found by us. Although pharmacists and nurses were not trained on the use of POx as they are not allowed to provide health care unsupervised by a medical doctor, the POx was used by these provider types and hypoxemia was relatively higher in the proportions consulted by these provider types. This inter-provider variation in the proportion of hypoxemic children could be due to incorrect use of the device, or to true variation in the distribution of cases. Anthropometric parameters (height, weight and middle-upper arm circumference) and vital signs (temperature, respiratory rate, pulse rate and blood pressure) were not routinely measured and immunization status could not be assessed as most of the caregivers did not carry the immunization card with them during the consultation visit. Since this was a pragmatic study, health system strengthening, specifically related to completion of referrals was not a part of this, which could likely to have affected referral completion.

The prevalence of hypoxemia among sick children presenting to rural health facilities of northern India was low but was associated with a high mortality risk. However, only less than one-third of hypoxemic sick children were referred by health workers and only one in five of them actually completed referral. Therefore, there is an urgent need to identify and address factors associated with low completion of referrals as well as mechanisms and approaches allowing for effective referral mechanisms, including if appropriate counter-referral to primary care services for follow-up.

## Contributors

SA, HL, FB, MR, MEF, FB, FS, KW and VDA were responsible for conceptualisation of the study. SA, HL, GAL, SC and FB were responsible for the methodology of the study. SA, DK, AJ and MA were responsible for the implementation of the study in India. MR, MEF, KS and GK were responsible for the intervention implementation, with support from other members of the TIMCI collaborator group. Data was collected by KGMU research assistants, overseen by SA, DK, AJ and MA. Data management was led and coordinated by HL globally and by DK and AJ, in India. SC developed the statistical analysis plan with inputs from SA, DK, AKP, GGA, AJ, HL, GAL, FB, KW and VDA. AKP and GGA performed the statistical analysis with inputs from SA, DK, MA, AJ, KS, HL, GAL, GK, SC, MR, MEF, FB, FS, KW and VDA. Interpretation of findings was conducted collectively by all the authors. The original draft of the manuscript was prepared by SA, DK and AKP with review and feedback by all other authors. Overall oversight was by Principal Investigators KW and VDA globally and SA in India. FS was responsible for research activity planning and execution globally, with DK and MA in India. KW, VDA and SA and other members of the wider TIMCI collaborator group were responsible for acquiring funds. The full list of the TIMCI collaborator group is available here: https://zenodo.org/communities/timci/about.

## Data sharing statement

The dataset will be publicly available in the Zenodo repository after the main TIMCI RCT publication (https://zenodo.org/records/14024887).

## Declaration of interests

The authors declare no competing interests.
