# Supplementary material for: Prevalence of hypoxemia among sick children, aged under-five years, seeking healthcare at primary health facilities in Uttar Pradesh, India: an observational-cohort study
Source: Lancet Reg Health Southeast Asia. 2025 Dec 15;43:100706. doi: 10.1016/j.lansea.2025.100706 (PMC12766599; doi:10.1016/j.lansea.2025.100706)
Supplement: Supplementary Table [file mmc1.pdf]

Supplementary material

Table of contents

Serial Number	Description	Page Number
1	Supplementary picture 1: TIMCI Consultation card	2
2	Supplementary Table 1: Distribution of presenting symptoms on basis of types of healthcare providers.	3

Supplementary picture 1: TIMCI Consultation card

TIMCI Consultation Card					
To be filled by Research Assistant					
Date: __ __ /__ __/__ __ __ __					
**1**	Name of child				
**2**	Completed age	__ __ (years) __ __ (months)			
**3**	Gender (✓ appropriate)	[ ] Male [ ] Female [ ] Other			
**4**	Father/Mother Name				
To be filled by attending Doctor					
5	**Examination**				
**5.1**	Height	__ __ __ cms.	**5.2**	Weight	__ __.__ Kgs
**5.3**	Temp.	__ __ __.__ °F	**5.4**	Spo2	__ __ __ %
**5.5**	Respiratory rate	__ __ __/min			
**6**	Diagnosis				
7	Medicines / Treatment advised<br>*(Please indicate "**Inj**" for injectables)*				
	Injectables			Oral/others	
**7.1**			**7.5**		
**7.2**			**7.6**		
**7.3**			**7.7**		
**7.4**			**7.8**		
**8**	Lab test advised? (✓ appropriate)	[ ] Yes [ ] No			
**9**	Management (✓ appropriate)	[ ] Out patient treatment<br>[ ] Advised Follow-up<br>[ ] Referred to higher facility<br>[ ] Admitted in IPD			

**Supplementary Table 1: Distribution of presenting symptoms on basis of types of healthcare providers.**					
Healthcare provider type	Presenting Symptoms				
	Any Danger Sign	Difficult Breathing	Fever	Cough	Diarrhea
	m/n (%)				
Pediatrician	53/2596 (2·0)	18/2596 (0·7)	1506/2596 (58·0)	1338/2596 (51·5)	276/2596 (10·6)
MBBS	446/13229 (3·4)	79/13229 (0·6)	8915/13229 (67·4)	5803/13229 (43·9)	607/13229 (4·6)
AYUSH	169/4809 (3·5)	71/4809 (1·5)	3276/4809 (68·1)	1986/4809 (41·3)	229/4809 (4·8)
Pharmacist	39/1841 (2·1)	7/1841 (0·4)	1277/1841 (69·4)	830/1841 (45·1)	92/1841 (5·0)
Nurse	23/1085 (2·1)	1/1085 (0·1)	756/1085 (69·7)	455/1085 (41·9)	18/1085 (1·7)

Abbreviations: MBBS: Bachelor of Medicine and Bachelor of Surgery; AYUSH: Ayurveda, Yoga and Naturopathy, Unani, Siddha and Homeopathy.
